# Reanalysis of RNA-Sequencing Data Reveals Several Additional Fusion Genes with Multiple Isoforms

**DOI:** 10.1371/journal.pone.0048745

**Published:** 2012-10-31

**Authors:** Sara Kangaspeska, Susanne Hultsch, Henrik Edgren, Daniel Nicorici, Astrid Murumägi, Olli Kallioniemi

**Affiliations:** Institute for Molecular Medicine Finland (FIMM), Helsinki, Finland; The Institute of Cancer Research, London, United Kingdom

## Abstract

RNA-sequencing and tailored bioinformatic methodologies have paved the way for identification of expressed fusion genes from the chaotic genomes of solid tumors. We have recently successfully exploited RNA-sequencing for the discovery of 24 novel fusion genes in breast cancer. Here, we demonstrate the importance of continuous optimization of the bioinformatic methodology for this purpose, and report the discovery and experimental validation of 13 additional fusion genes from the same samples. Integration of copy number profiling with the RNA-sequencing results revealed that the majority of the gene fusions were promoter-donating events that occurred at copy number transition points or involved high-level DNA-amplifications. Sequencing of genomic fusion break points confirmed that DNA-level rearrangements underlie selected fusion transcripts. Furthermore, a significant portion (>60%) of the fusion genes were alternatively spliced. This illustrates the importance of reanalyzing sequencing data as gene definitions change and bioinformatic methods improve, and highlights the previously unforeseen isoform diversity among fusion transcripts.

## Introduction

Gene fusions represent a well-established class of mutations in hematological diseases and sarcomas, with the *BCR-ABL* fusion in chronic myeloid leukemia [1], [2] and different *EWS*-fusions in sarcomas [3] being prototype examples with diagnostic, prognostic and therapeutic value [4]. Recent discoveries of recurrent fusion genes in lung and prostate cancers indicate that gene fusions may be more prevalent even in solid tumors [5]–[7]. Unlike in prostate cancer where the *TMPRSS2-ETS*–family translocations are found in ca. 79% of the tumors, there have been no common breast cancer fusions discovered. *ETV6-NTRK3* in secretory breast ductal carcinoma [8], *MYB-NFIB* in adenoid cystic carcinoma of the breast [9], and the recently discovered *MAST*- and *NOTCH*-fusions [10] are recurrent, but still infrequent. Compared to fluorescent *in situ* hybridization, spectral karyotyping or cytogenetic techniques applied for fusion gene discovery, massively parallel sequencing now allows much more sensitive and specific fusion gene detection [11], [12], and has already increased the number of gene fusions reported also in breast cancer. Particularly RNA-sequencing (RNA-seq) that permits direct detection of expressed fusion transcripts, provides a method for fusion gene detection largely independently of the genomic complexity of many cancers. In addition to the next generation sequencing technologies, a stratified bioinformatic analysis pipeline is essential for effective fusion gene detection, including separation of false positive fusions. All recent reports on breast cancer fusion gene detection rely on paired-end RNA-seq data coupled with a fusion gene detection methodology, which incorporates steps for filtering of false positives and methodological and biological noise [10], [13]–[19]. We recently used paired-end RNA-seq and a bioinformatic fusion gene discovery pipeline building upon a tiling pattern of sequencing short reads on the transcript fusion-fusion junctions. Utilizing this strategy, integrated with high-resolution chromosomal copy number analysis, we discovered 24 novel breast cancer specific fusion genes, the majority of which were located at copy number transitions or within or in close proximity to high-level DNA amplifications [19]. Here, we refine the bioinformatic prediction of fusion events by reanalyzing the paired-end RNA-seq raw data using an updated Ensembl annotation and by allowing several partner genes per fusion. Using this approach, we bioinformatically identify and experimentally validate 13 additional fusion genes, of which 12 were previously predicted or reported [16]–[18], [20] and one is entirely novel, highlighting the importance of frequent fine-tuning of bioinformatic methods for fusion gene discovery. Moreover, we demonstrate that many of the discovered fusion genes are present in multiple transcript isoforms, underlining a previously unanticipated complexity among gene fusions.

## Results

### Identification and validation of fusion transcript candidates

**Figure 1:**
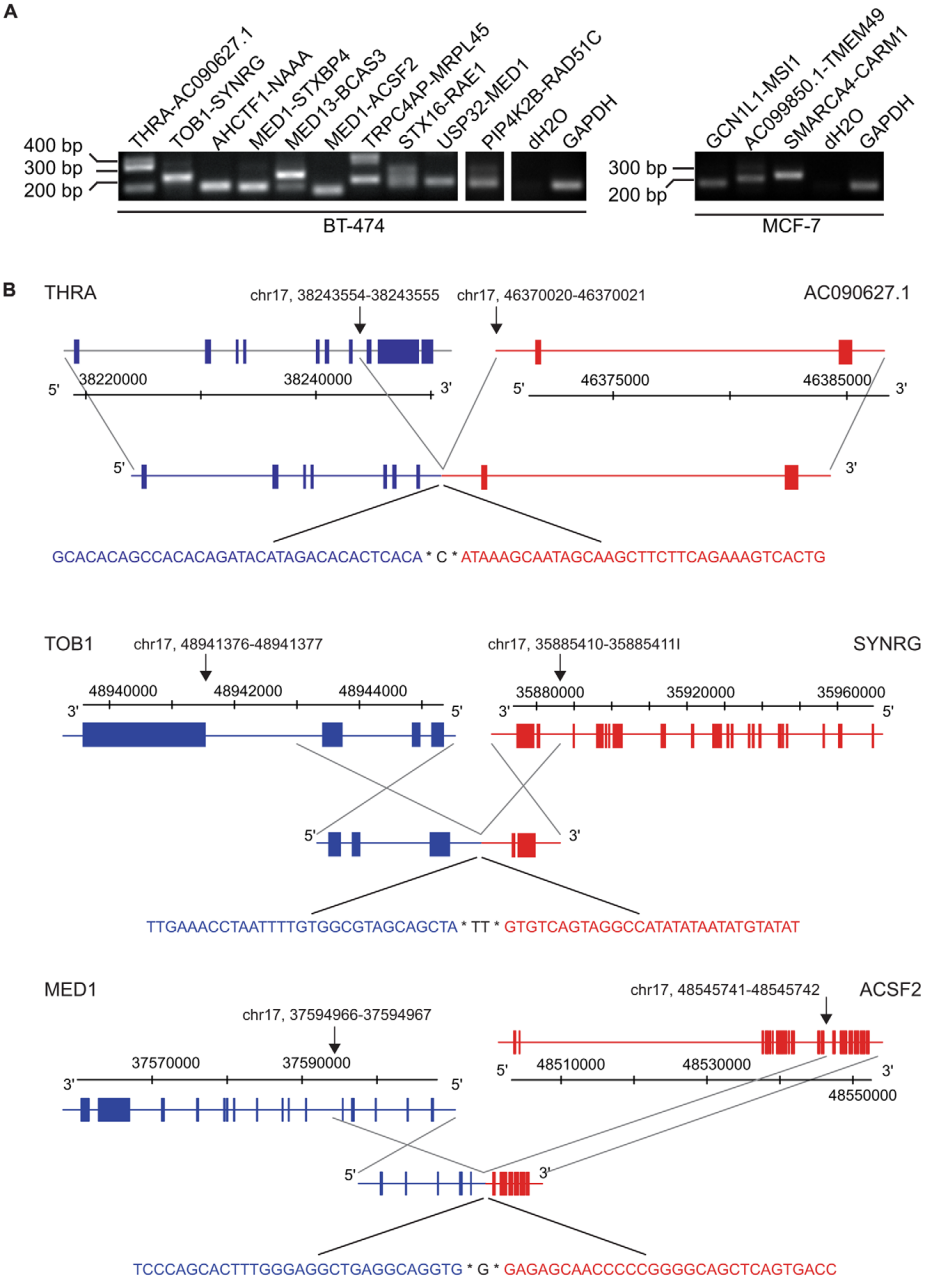
Transcript- and genomic level validation of the fusion genes. A. Validation of the fusion genes from BT-474 and MCF-7 on the cDNA level by RT-PCR. B. Genomic DNA sequence at the fusion gene break point of *THRA-AC090627.1* (top), *TOB1-SYNRG* (middle) and *MED1-ACSF2* (bottom). Chromosomal positions of the fusion break points are indicated by black arrows. Gene and transcript structures as well as nucleotide sequences at the break points are drawn in blue for 5′ and in red for 3′ partner genes. Gene structures above and below chromosome coordinates imply forward and reverse strand, respectively. Transcript structures of gene fusions are indicated below the gene structures and connected with gray lines. Genomic DNA sequence at the break point (indicated by asterisk) is shown below the transcript structures. Black color indicates nucleotides that match to both (*THRA-AC090627.1, MED1-ACSF2*) or neither partner genes (*TOB1-SYNRG*).

We recently described a bioinformatic strategy for discovery of fusion genes from RNA-seq data. Using this fusion gene pipeline, we predicted and validated 24 novel and 3 previously known fusion transcripts, some of them potentially conveying growth advantage to the cancer cells [19]. To further improve the bioinformatic prediction we reanalyzed the RNA-seq raw data according to the workflow described previously. This included selection of paired-end reads of which the ends align to two separate genes, followed by alignment of short reads against all possible exon-exon junctions of the transcriptome, and looking for a tiling pattern of the short reads across the exon-exon junctions. Additionally, we now included two major modifications to the prediction pipeline: 1) usage of an upgraded version of Ensembl (www.ensembl.org) for mapping the exact exon-exon junction of the two fusion partner genes, and 2) allowing more than one fusion gene partner per fusion gene. Using these criteria, we identified 16 new fusion transcript candidates, of which two were ultimately excluded as false positives most likely owing to high expression levels of the individual fusion partner genes, and one was classified as a read-through transcript (Table 1, Table S1). Of the remaining 13 fusion gene candidates, eight were predicted to code for *in-frame* fusion transcripts (Table 1), and all 13 were subsequently validated by RT-PCR across the exon-exon boundaries of the fusion transcripts followed by Sanger sequencing (Figure 1A). However, it is possible that some of the fusions that were predicted to be *out-of-frame* actually do retain an intact open reading frame through alternative splicing, as they would otherwise be subject to recognition and degradation by nonsense-mediated mRNA decay (and hence not detected on the mRNA-level). We next addressed the presence of genomic DNA rearrangements underlying the fusion transcript formation by conducting genomic DNA sequencing across the anticipated break points of three selected fusions. This approach revealed the exact genomic break point for *THRA-AC090627.1*, *TOB1-SYNRG* and *MED1-ACSF2* (Figure 1B and Table S2). In *THRA-AC090627.1* both partners were found to be fused together in their 5′–3′ orientation, whereas in *TOB1-SYNRG* both genes and in *MED1-ACSF2* only the 5′ partner gene *MED1* are inverted (Figure 1B). Genomic changes are likely to act as foundations for most of the fusion transcripts, as mRNA *trans*-splicing although possible, has been only rarely documented in vertebrates [21].

**Table 1:** Identified and validated fusion gene candidates.

Sample	5′ gene	5′ chromosome	3′ gene	3′ chromosome	N. paired- end reads	N. junction reads	In-frame	Amplified	On a genomic break point	Previous characterization
BT-474	*THRA*	17	*AC090627.1*	17	77	38	yes	no	yes	bioinformatic prediction [18], validation [20]
BT-474	*TOB1*	17	*SYNRG*	17	25	38	yes	no	yes	bioinformatic prediction [18]
BT-474	*AHCTF1*	1	*NAAA*	4	20	11	yes	yes	yes	bioinformatic prediction [17]–[18], validation [17]
BT-474	*MED1*	17	*STXBP4*	17	11	13	yes	yes	no	bioinformatic prediction [18]
BT-474	*MED13*	17	*BCAS3*	17	9	3	no	yes	yes	bioinformatic prediction [18]
BT-474	*MED1*	17	*ACSF2*	17	8	10	no	yes	yes	bioinformatic prediction [18]
BT-474	*TRPC4AP*	20	*MRPL45*	17	5	3	yes	yes	no	bioinformatic prediction [17]–[18], validation [17]
BT-474	*STX16*	20	*RAE1*	20	4	8	no	yes	yes	bioinformatic prediction [18]
BT-474	*USP32*	17	*MED1*	17	4	3	no	yes	borderline	no
BT-474	*PIP4K2B*	17	*RAD51C*	17	3	3	no	yes	yes	bioinformatic prediction [17], validation [17]
MCF-7	*GCN1L1*	12	*MSI1*	12	3	2	yes	no	borderline	identification with RNA-PET, validation [16]
MCF-7	*AC099850.1*	17	*TMEM49*	17	4	6	yes	yes	yes	bioinformatic prediction [18]
MCF-7	*SMARCA4*	19	*CARM1*	19	3	2	yes	yes	yes	identification with RNA-PET, validation [16]

13 fusion genes were detected from BT-474 and MCF-7 breast cancer cells. A minimum of two paired-end reads and two fusion junction spanning reads were a prerequisite for choosing a fusion gene candidate for further analysis. Copy number amplification, location on a genomic break point (at least one of the fusion partner genes in both cases) and *in-frame* prediction are indicated. Lower level copy number gains were not included in the analysis. “Previous characterization” -column summarizes the level of information available for the individual fusion transcripts prior to this study. Validation refers to verification at the transcript level by RT-PCR or Northern blotting (for [20]).

### Genomic rearrangements underlie majority of fusion events

**Figure 2:**
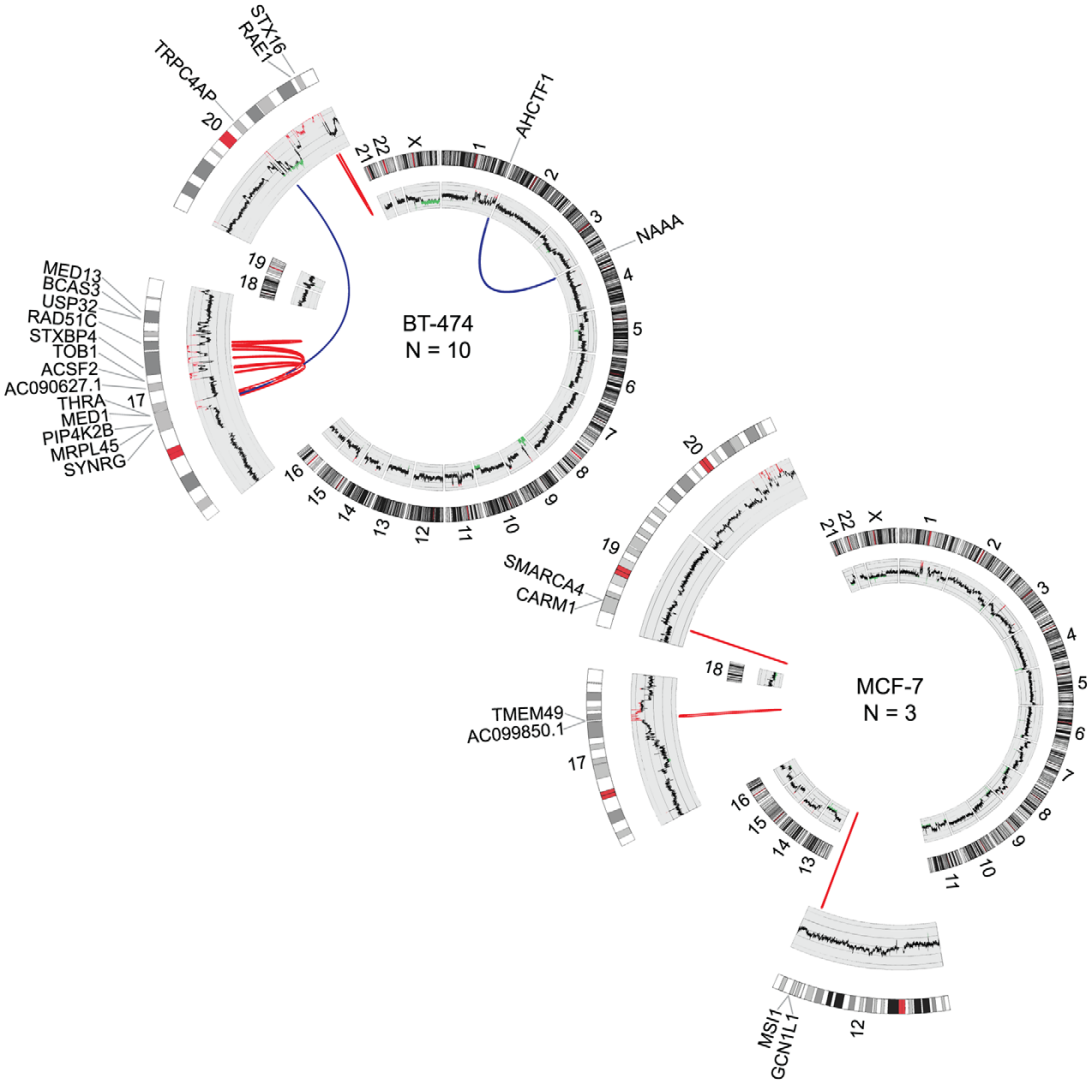
Genomic rearrangements underlying fusion gene formation. Circos plots illustrating chromosomal translocations in BT-474 (upper) and MCF-7 (lower). Chromosomes are drawn into scale around the rim of the circle and data are plotted on these coordinates. Intrachromosomal (red) and interchromosomal (blue) fusions are indicated by arcs. Copy number profiles are plotted in the inner circle. Amplifications are shown in red and deletions in blue. N denotes the number of fusion genes per cell line.

**Figure 3:**
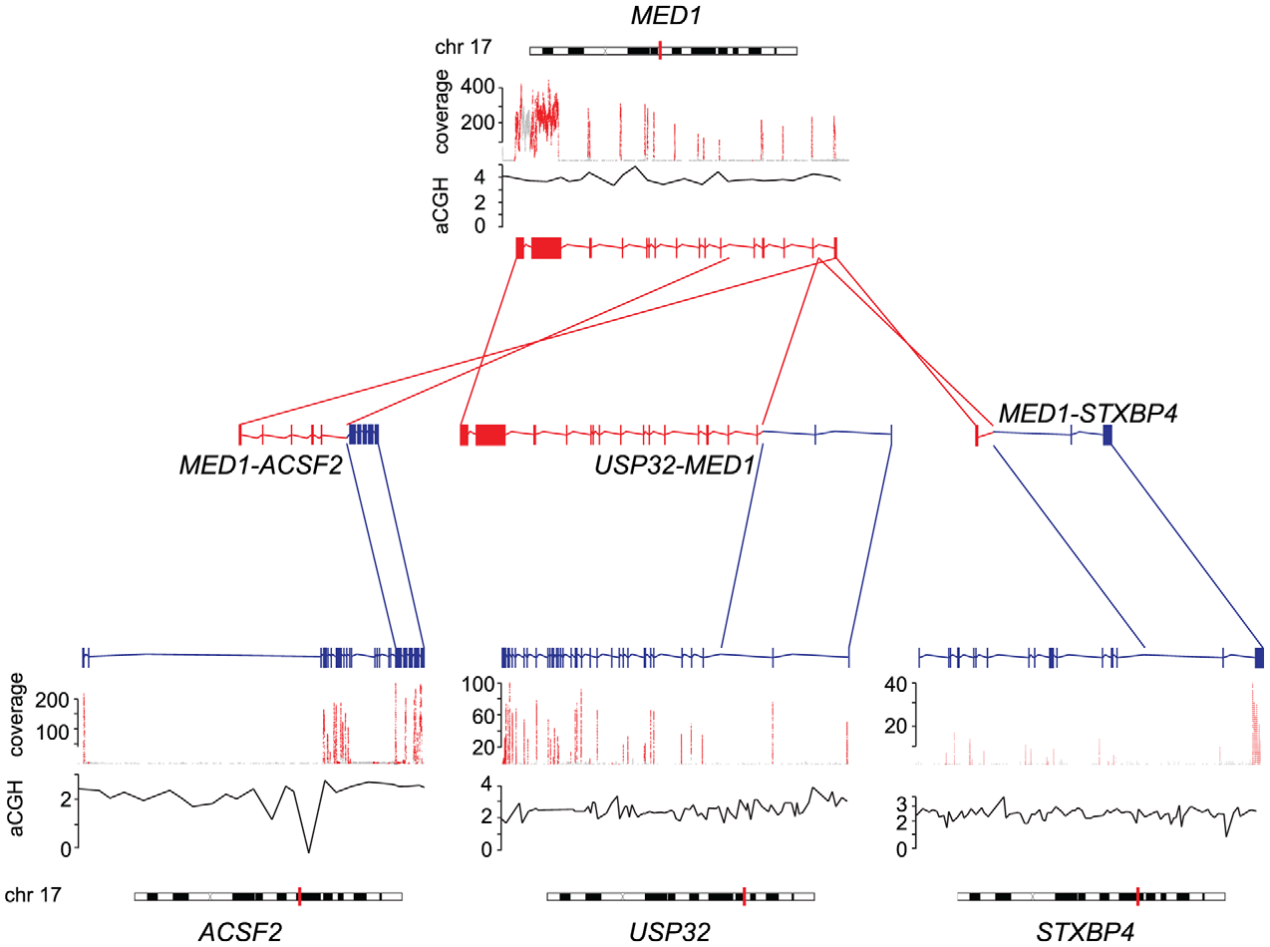
MED1 forms fusions with several partner genes. Exonic expression of *MED1* and its partner genes *ACSF2*, *USP32* and *STXBP4* is indicated by sequencing coverage (red). Copy number changes measured by aCGH (black lines) in reference to normal copy number (horizontal gray lines), and chromosomal positions (vertical red lines on chromosomes) are indicated. Transcript structures of wild type genes as well as gene fusions are indicated by red (*MED1*) and blue (*ACSF2, USP32, STXBP4*), and connected with lines of same color. Arrows show the 5′ 3′ direction of the genes.

As previous studies by us [19] and others [22], [23] have shown that fusion genes frequently occur at DNA copy number transition points, we analyzed the RNA-seq data in conjunction with array CGH (aCGH), and examined the copy number profiles of BT-474 and MCF-7 cells at the fusion gene locations. In agreement with previous findings, the vast majority of the fusion gene partners under study (11/13) were located at genomic break points (Table 1, Figure 2). Moreover, over half of the fusion genes (8/13), were located at high level amplicons at 17q and 20q including fusions such as *MED13-BCAS3*, *TRPC4AP-MRPL45*, *STX16-RAE1* and *AC099850.1-TMEM49* (Table 1, Figure 2). From the BT-474 and MCF-7 fusion genes described recently by us, and from the ones reported here, 70% resided in these high level amplicons and were therefore not a result of classical balanced rearrangements (Figures S1 and S2). In fact, only one of these fusions, *PPP1R12A-SEPT10*, was not associated with any type of a detectable copy number break point, nor was it located in the proximity of amplified genomic regions [19]. Three of the fusion gene partners (*MED1, BCAS3, TMEM49*) and one we identified previously (*RPS6KB1*), were fused to more than one partner gene (Table 1, Figure 2, [19]). For example, *MED1*, which is not located at a genomic break point, but resides within a high level amplification on chromosome 17q, was found fused to another partner gene in an amplified region (*STXBP4* in *MED1-STXBP4*) but also to two other genes that are close to copy number break points, but not amplified (*ACSF2* in *MED1-ACSF2* and *USP32* in *USP32-MED1*, Figure 3). All these *MED1*-fusions occur within the same cell line, BT-474. In two of the *MED1*-fusions the partner genes are located on opposite strands, implying inversion, whereas in one of the fusions the partner genes are on same strand, further emphasizing the complex nature of fusions involving promiscuous gene partners and the genetic rearrangements underlying them (Figure 3).

### Structural characteristics and expression of the fusion genes

**Figure 4:**
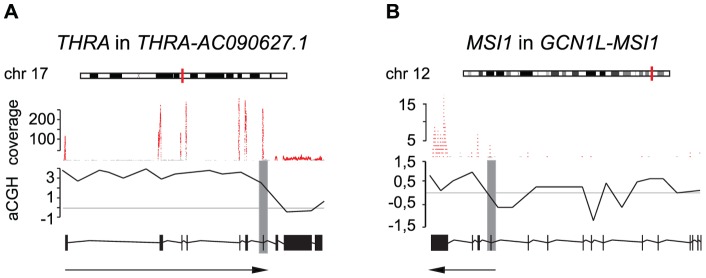
Exclusive expression of fusion partner genes. Exonic expression of *THRA* in *THRA-AC090627.1* (A) and *MSI1* in *GCN1L-MSI1* (B) is indicated by sequencing coverage (red). Copy number changes measured by aCGH (black lines) in reference to normal copy number (horizontal gray lines), chromosomal positions (vertical red lines on chromosomes) and fusion break points (vertical gray bars) are shown. Transcript structures are indicated below the aCGH profiles with the arrows pointing to the parts of the transcripts taking part in the fusions.

Examination of the genomic structures of the fusion gene set revealed that ca. two-thirds of them fall into the category of promoter-coding fusions (i.e. fusions between the promoter sequences of the 5′ fusion partner and the coding sequences of the other fusion partner). Correspondingly, 2/13 and 3/13 were coding-3′UTR and coding-coding fusions, respectively (Table S3). Irrespective of the type of the fusion transcript (whether promoter-coding, coding-3′UTR or coding-coding fusion), a majority of them were truncating mutations where neither gene partner is included in its entirety in the fusion, which is in line with previous research [14]. In some cases the coding sequences of the 5′ gene are truncated (e.g. *THRA-AC090627.1*), whereas in others the coding sequences of either both of the fusion partners or of the 3′ gene are disrupted (e.g. *PIP4K2B-RAD51C*, *GCN1L1-MSI1*) (Table S3). In addition to noting the structural classes of the fusion genes, we sought to deduce the transcriptional consequences of these by combining the copy number data with the sequencing coverage of different fusion partner genes. In some cases, exclusive expression of the fusion genes compared to their wild type partner genes was seen. In *THRA-AC090627.1*, a genetic rearrangement visible as an aCGH copy number change juxtaposes the exons 1–7 of *THRA* to exon 2 and 3′UTR of *AC090627.1* on chromosome 17. Similarly, disruption of *GCN1L1* after exon 2 places the 5′UTR and exons 1–2 of this gene in front of exons 12–15 of *MSI1* on chromosome 12 (Figure 4A and B). In both cases, these rearrangements result in exclusive expression of the exons of *THRA* and *MSI1* taking part in the fusion. This kind of expression pattern can be indicative of functional relevance of the fusion in activating the otherwise not expressed parts of the partner genes involved [19].

### Several fusion transcripts have multiple splice variants

**Figure 5:**
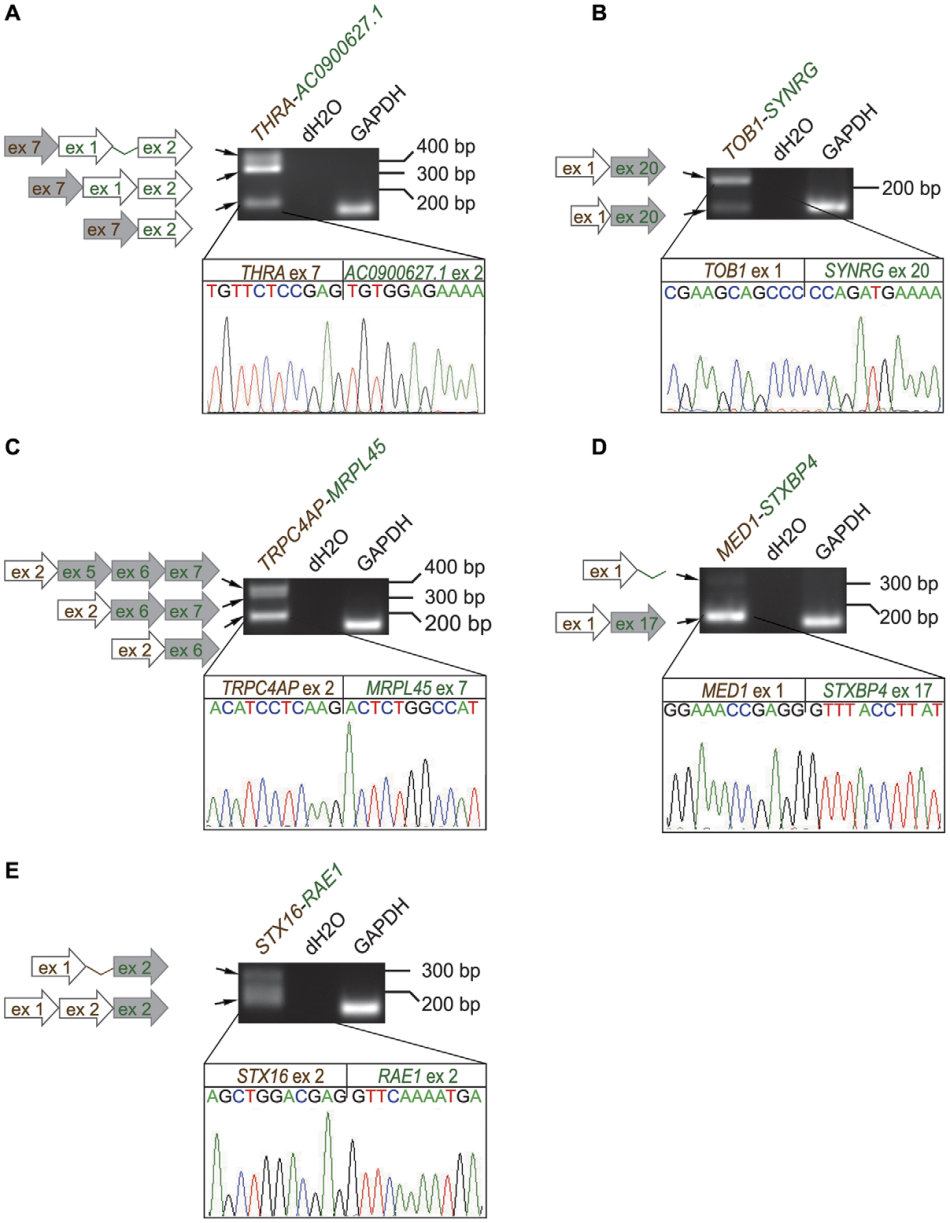
Several fusion transcripts have multiple splice variants. Five examples from BT-474 (A. *THRA-AC090067.1*, B. *TOB1-SYNRG*, C. *TRPC4AP-MRPL45,* D. *MED1-STXBP4* and E. *STX16-RAE1*) are presented. Multiple splice variants are visible as RT-PCR bands, and schematically represented by the arrows to the left. Chromatograms show the actual cDNA sequence break points of the main predicted fusion isoforms, and are connected with lines to the corresponding RT-PCR bands. Gray arrows  =  coding sequence, white arrows  =  untranslated exon or 3′/5′ UTR, thin lines connecting exons  =  intronic regions. 5′ partner genes are represented by brown color, 3′ partner genes by green.

RT-PCR analysis followed by Sanger sequencing revealed that 8/13 fusion genes were expressed as multiple isoforms, some with as many as three to four distinct splice variants (Figure 5). For example for *THRA-AC090627.1,* where the coding sequences of *THRA* were fused to the 3′UTR of *AC090627.1,* three isoforms with either one or two untranslated exons of *AC090627.1* were discovered, with or without retention of the intervening intron (Figure 5A). In other cases, isoforms with either an intact or a truncated exon of one of the fusion partners were present (*TOB1-SYNRG*, *AC099850.1-TMEM49*, Figures 5B and S3). Interestingly, in some cases the sequence variation of the different individual fusion isoforms is reflected in the functional domains coded by them. For example, in the promoter-coding fusion *TRPC4AP-MRPL45* the smallest isoform comprising exons six and seven of *MRPL45* is predicted to code for a low complexity protein (i.e. containing little amino acid diversity). In contrast, the two largest isoforms with exons five-seven and six-seven of *MRPL45* present, are predicted to encode a TIM44 domain involved in translocation of proteins across the mitochondrial membrane (Figure 5C, Table S3). In some of the fusion isoforms, intron retention was seen. Examples include *MED1-STXBP4* and *STX16-RAE1* where intronic sequence was brought by the 3′ partner, and the 5′ partner, respectively (Figure 5D-E). This adds a previously underestimated level of sequence diversity to fusion genes on the transcript level.

## Discussion

In the present study, we report the identification of several breast cancer fusion transcripts by refining our recently described bioinformatic fusion gene discovery pipeline [19]. Together with the previous publication, we have therefore identified a total of 40 fusion genes, which is, to our knowledge, along with the recent work from the Chinnaiyan group [10] the highest number of breast cancer gene fusions identified from the same experimental set-up. Importantly, all the genes we have identified, have also been experimentally validated and found to be specific to the sample where the RNA-seq experiment suggested them to be present. Although in recent years there has been a growing number of reports on identification of fusion genes in solid tumors [24], [25], including breast cancer [10], [13]–[18], fusion gene detection has been complicated by the high false positive rate [13], [26]–[29]. In our experience, the most indicative feature of a true fusion is the tiling pattern of the short reads running across the exon-exon boundary of the fusion gene. In these cases, as little as two paired-end reads and two junction covering short reads are supportive of a true fusion (Table 1) [19].

In this study, we sought to address the effect of two bioinformatic steps on fusion gene detection: 1) updates in the annotation database Ensembl, and 2) commission of more than one fusion partner per gene per sample. As gene annotation is continuously evolving, updates in the annotation databases yield additional fusion genes, as demonstrated here by us. By allowing more than one fusion partner, we sought to identify possible indiscriminate fusion genes among our candidate gene fusions. Based on our data, fusion partners that can recombine with several distinct partner genes are often found in breast cancer samples. Some well-established fusion genes have also been shown to be promiscuous, examples including *MLL-* in leukemias, *EWS-* in sarcomas, *RET-* in carcinomas and *TMPRRS2-* and *ETV1-*fusions in prostate cancer [4], [7], [30], [31]. We found promiscuous fusion gene partners within the same sample, possibly reflecting the more rearranged genomes of cancer cell lines, whereas the different *MLL-*, *EWS-* etc. fusions occur one per sample, with diversity in fusion partners between the samples. We found *BCAS3* fused with two different 5′ partners (*BCAS4-BCAS3*, *MED13-BCAS3*), *MED1* with two separate 3′ genes (*MED1-STXBP4*, *MED1-ACSF2*) and a 5′ gene (*USP32-MED1*), and *TMEM49* was the 3′ partner fused to *AC099850.1* as well as *RPS6KB1* (Table 1, [19]). Some of these partner genes have recently also been documented by others as having several fusion partners [16], [17]. One of the most intriguing fusion partners is *RPS6KB1*, which we found fused to *SNF8* and *TMEM49* (*RPS6KB1-SNF8*, *RPS6KB1-TMEM49*), and that has been found in a subset of clinical breast cancer samples harboring 17q23-chromosome amplification, albeit with structural heterogeneity [16]. Hence, *RPS6KB1* may function both as a recurrent and promiscuous fusion gene partner in a subset of breast cancers. Interestingly, both *MED1* and *TMEM49* are also amplified genes, and thus, it could be speculated that repeated chromosome breaks occurring in the same gene during amplicon formation (e.g. during breakage-fusion-bridge cycles) could pave the way for the same gene to form many fusions with different partner genes.

An emerging theme with fusion transcripts is their presence at genomic copy number transitions, and moreover, at high-level amplicons [22], [23]. We carried out aCGH in order to create a genomic map with roughly 2 kb resolution [32] to study the chromosomal break points underlying structural fusion-generating rearrangements. From the 40 fusion genes described by us here and previously [19], the majority (60%) were associated with gene amplifications (Figures S1 and S2). Interestingly, whereas balanced genetic rearrangements (albeit often with microdeletions) prevail in hematopoietic diseases, this seems not to be the case for the gene fusions discovered in solid tumors [31]. Another structural feature of fusion genes appears to be the prevalence of intrachromosomal fusions over interchromosomal ones. Furthermore, there is a predominance of promoter-donating fusions over the coding-coding and coding-3′UTR ones, which is in line with recent research [17], [22]. This raises the question about possible genomic mechanisms and biological drivers of fusion gene formation. In a network analysis of known fusion genes in cancer, three separate hubs were found, involving mainly transcription factors and tyrosine kinases pointing to a non-random nature of fusion gene formation [4]. Others have suggested a more indiscriminate nature of the process involving, for example, spatial proximity of the partner genes in the interphase chromosomes within the nucleus. Also, movement of genes on different chromosome loops into the same transcription factories has been proposed [22]. However, these methods may apply better to leukemic fusion genes, which are less likely to involve copy number changes, such as high-level DNA amplifications. Chromothripsis, chromosome shattering in a spatially confined region, can also lead to rearrangements, and has been documented for example in colorectal cancer [33]. At the sequence level, a small deficit of CG nucleotides, and in some cases sequences of overlapping microhomology have been documented at the rearrangement points [22]. These facts could be indicative of genomic instability and non-homologous end-joining being active in fusion gene formation. Indeed, here we observe that the sequenced DNA stretches few hundred base pairs around the genomic fusion break point are very AT-rich for two out of three examined fusions; the AT-content being 60% for *THRA-AC090627.1* and 75% for *TOB1-SYNRG* (Figure 1B and Table S2). In the immediate vicinity of the fusion junction short stretches of identical sequence, just few nucleotides long, can be seen on both sides of the break. For *TOB1-SYNRG*, also a two-nucleotide long non-templated sequence is found at the fusion junction (Figure 1B and Table S2). These findings are in line with previous descriptions on nucleotide-level break point compositions [22]. Most likely, the process of fusion generation is influenced by a variety of both genomic mechanisms and the potential clonal advantage or disadvantage for cell growth and survival, which both act in a context-dependent manner.

Here we discovered that most of the fusions display transcript variants (ca. 60%, 8/13), which is more than previously anticipated (Figures 5, S3, S4, Table S3). Before just a handful of breast cancer fusion genes were reported to be alternatively spliced by us [19] and three additional studies [16]–[18]. Furthermore, here we observed transcript level retention of intronic sequences in the gene fusions (e.g. *MED1-STXBP4*, *STX16-RAE1*, Figure 5), adding yet another level of complexity to the fusion gene structure. As most fusion break points occur in introns, the transcriptional machinery is forced to switch to another exon or alternatively to acquire a new acceptor splice site in the intron where the breakage and fusion happen, in order to produce an *in-frame* transcript variant. Indeed, intron retention in some of the transcript variants would indicate that this does not always occur. Whether all the fusion transcript variants produce *in-frame* protein products with potentially distinct functional domains remains to be elucidated.

In conclusion, in recent years the rapid progress in next generation sequencing technologies has led to the concordant development of bioinformatic approaches for mining the raw sequencing data. We and others have exploited RNA-seq for the discovery of fusion genes [13]–[15], [18], [19], [34], [35]. Here, we demonstrated the need for review and development of bioinformatic fusion gene pipelines and by doing so, discovered and experimentally validated several breast cancer fusion genes. This emphasizes the importance of continuous re-evaluation of the bioinformatic methods to predict fusion genes. Furthermore, our data revealed that many of the fusion genes are expressed in several transcript isoforms, highlighting a previously unanticipated level of complexity in the fusion gene build-up. Even if the majority of fusion genes discovered in solid tumors are present at very low frequency or are private events, they may still contribute to the etiology and progression of the individual tumors. The roles of the individual fusion isoforms in these processes remain to be determined.

## Materials and Methods

### Cell culture

BT-474 and MCF-7 cells were obtained from American Type Culture Collection. KPL-4 was a kind gift from Dr. Junichi Kurebayashi, Department of Breast and Thyroid Surgery, Kawasaki Medical School, Japan [36]. BT-474 cells were grown in DMEM with L-Glutamine (PAN Biotech) supplemented with 10% FCS (Gibco), 0,1% bovine insulin (Sigma) and 1% penicillin/streptomycin (Gibco). MCF-7 and KPL-4 cells were grown in DMEM (Euro Clone) supplemented with 10% FCS (Gibco), 5% L-Glutamine and 1% penicillin/streptomycin. All cell lines were cultured at 37°C with 5% CO2. Total RNA was isolated with TRIzol Reagent (Invitrogen) according to the manufacturer's protocol, when the cells were ca. 80% confluent.

### Paired-end RNA-sequencing

For fusion gene detection data from the previously produced paired-end RNA libraries of the BT-474, MCF-7 and KPL-4 cell lines were used [19]. Briefly, messenger RNA was isolated with oligo-dT Dynabeads (Invitrogen) and then fragmented to an average size of 200 nt. 1 µg of mRNA was then synthesized into double stranded cDNA. The 3′ and 5′ overhangs of the templates were repaired with T4 DNA polymerase, Klenow DNA polymerase and T4 PNK (New England BioLabs). Before the paired-end adaptors were ligated with Ultrapure DNA ligase (Enzymatics) or quick DNA ligase (New England BioLabs), an additional A-base was added to the template by using the Klenow 3′ to 5′ exo –enzyme (New England BioLabs). The paired-end libraries were size selected and amplified using the Pfx polymerase (Invitrogen). The libraries were sequenced with the 1G Illumina Genome Analyzer 2X (Illumina).

### Fusion gene detection and characterization

Throughout this study the sequences were aligned to the human genome using the Ensembl version 61 as previously described [19]. The fusion gene detection pipeline was built and employed as follows. First, short reads aligning to rRNA, mtRNA or other contaminant sequences (e.g. adaptors) are filtered out. The filtered short reads are then grouped into 1) not aligning 2) uniquely aligning and 3) aligning to multiple loci on the genome. Here, the alignment was done with the Bowtie software version 0.11.03, allowing a maximum of 3 mismatches [37]. A read is considered to align uniquely if there is a single best alignment against the reference. The reads which map uniquely on the genome and the reads which do not map on the genome are further mapped to the transcriptome in such a way that all alignments are reported. In this step no distinction between unique and multiple mappings of the same read is made in order to take into consideration that the same exon can appear in several transcripts. All alignments are analyzed as follows. First, all reads which map simultaneously on different transcripts from different genes are used to build a list of potentially-similar-genes (from a sequence point of view) and also removed from further analyses. This filter is used to exclude gene-gene pairs that share stretches of high sequence similarity even though they are not classified as paralogs in any database. Second, a list of candidate fusion genes is built using the paired-read information and the mapping of reads on different transcripts belonging to different genes. The pairs of candidate fusion genes which (i) are in the list of potentially-similar-genes, or (ii) are adjacent genes (i.e. both genes are on the same strand and there is no other gene situated on the same strand between them), (iii) are paralogs of each other based on Ensembl version 61 or (iv) are supported by less than two paired-reads are removed from further analyses. The candidate exon pairs from two different genes are used to build an exon-exon database containing the exon junction sequences (splice site junctions) of all possible exon-exon combinations between each pair of genes. By aligning the reads that have not yet aligned anywhere against this database, the exon-exon fusion points are determined and the 5′ partner genes in the fusions defined. Additionally, more than one fusion partner per gene was allowed here, in contrast to [19], in which genes taking part in more than one fusion in the same sample were excluded. The raw sequencing data have been deposited in the NCBI Sequence Read Archive (SRA:SRP003186) previously [19]. For further characterization such as coverage of the fused genes and copy number changes, the sequencing data was integrated with the 1M oligo Agilent aCGH data as described previously [19]. Domain predictions of the fusion transcripts were done with the protein domain annotation resource SMART [38], [39].

### RT-PCR

For experimental validation the predicted fusion genes were Sanger-sequenced. First 4 µg of total RNA was transcribed to first strand cDNA by using the High Capacity cDNA Reverse Transcription Kit (Applied Biosystems) according to the manufacturer's protocol. Fusion gene specific primer pairs (Table S4) and Fast StartTaq DNA Polymerase (Roche) were utilized for the PCR reactions. The gel purified (GE Healthcare) PCR products were then cloned into pCR2.1-TOPO vector (Invitrogen). Sanger-sequencing of the clones with the ABI Prism 3730xl Sequencer (Applied Biosystems) confirmed the fusion transcript.

### Genomic DNA sequencing

To see if the fusion genes were genomically rearranged genomic DNA from the BT-474 cell line was isolated with the DNeasy Blood & Tissue kit (Qiagen). The fusions to be sequenced were chosen according to the following criteria and such that they would fairly represent the range of fusion genes identified: i) fusions that had previously been predicted solely by bioinformatic means (*TOB1-SYNRG, MED1-ACSF2*) as well as one that had also been validated at the transcript level (*THRA-AC090627.1*) (Table 1), ii) fusions which has several fusion partners (*MED1* in *MED1-ACSF2*), and iii) fusions where exome-sequencing data was available to guide the anticipated location of the genomic break point. PCR primers (Table S4) for amplification of DNA around the fusion break points were then designed based on visualization (The Integrative Genomics Viewer software, IGV, [40]) of exome-sequencing data of paired end reads mapping to each fusion partner. Genomic sequences were amplified with the Platinum Pfx DNA Polymerase (Invitrogen) for the *THRA-AC090627.1* fusion and with the Fast StartTaq DNA Polymerase (Roche) for *TOB1-SYNRG* and *MED1-ACSF2* fusions according to the manufacturers' instructions. The gel purified (GE Healthcare) PCR products were then Sanger-sequenced with the ABI Prism 3100xl capillary sequence analyzer (Applied Biosystems). The obtained sequences were aligned against the human genome using BLAT [41] with the “Near-exact matches” option. Sequences that aligned partially to both genes taking part in the fusion were used to locate the chromosomal breakpoints.

## Supporting Information

Figure S1 **Genomic rearrangements underlying fusion gene formation in BT-474.** Circos plot illustrating all chromosomal translocations in BT-474 reported by us here and previously [19]. Chromosomes are drawn into scale around the rim of the circle and data are plotted on these coordinates. Intrachromosomal (red) and interchromosomal (blue) fusions are indicated by arcs. Copy number profiles are plotted in the inner circle. Amplifications are shown in red and deletions in blue. N denotes the number of fusion genes per cell line.
(TIF)

Figure S2 **Genomic rearrangements underlying fusion gene formation in MCF-7.** Circos plot illustrating all chromosomal translocations in MCF-7 reported by us here and previously [19]. Chromosomes are drawn into scale around the rim of the circle and data are plotted on these coordinates. Intrachromosomal (red) and interchromosomal (blue) fusions are indicated by arcs. Copy number profiles are plotted in the inner circle. Amplifications are shown in red and deletions in blue. N denotes the number of fusion genes per cell line.
(TIF)

Figure S3 **Several fusion transcripts have multiple splice variants in MCF-7.** Transcript variants of MCF-7 fusion genes *GCN1L1-MSI1, SMARCA4-CARM1* and *AC099850.1-TMEM49* are presented. Multiple splice variants are visible as RT-PCR bands, and schematically represented by the arrows to the left. Chromatograms show the actual cDNA sequence break points of the main predicted fusion isoforms, and are connected with lines to the corresponding RT-PCR bands. Gray arrows  =  coding sequence, white arrows  =  untranslated exon or 3′/5′ UTR, thin lines connecting exons  =  intronic regions. 5′ partner genes are represented by brown color, 3′ partner genes by green.
(TIF)

Figure S4 **Several fusion transcripts have multiple splice variants in BT-474.** Transcript variants of BT-474 fusion genes *USP32-MED1, PIP4K2B-RAD51, AHCTF1-NAAA, MED13-BCAS3* and *MED1-ACSF2* are presented. Multiple splice variants are visible as RT-PCR bands, and schematically represented by the arrows to the left. Chromatograms show the actual cDNA sequence break points of the main predicted fusion isoforms, and are connected with lines to the corresponding RT-PCR bands. Gray arrows  =  coding sequence, white arrows  =  untranslated exon or 3′/5′ UTR, thin lines connecting exons  =  intronic regions. 5′ partner genes are represented by brown color, 3′ partner genes by green.
(TIF)

Table S1 False positive fusion transcript candidates. Three fusion gene candidates that passed the initial bioinformatic screen (supported by a minimum of two paired-end reads and two fusion junction spanning reads) were later experimentally proven to be false positives. Copy number amplification, location on a genomic break point (at least one of the fusion partner genes in both cases) and *in-frame* prediction are indicated. Lower level copy number gains were not included in the analysis.
(XLSX)

Table S2 Genomic sequences of fusion break points.
(XLSX)

Table S3 Several fusion transcripts code for functional protein domains. Type of fusion transcript (promoter-coding, coding-coding, coding-3′UTR) is indicated along with the sequences of the 3′ and 5′ fusion genes taking part in the fusion transcripts. Domain predictions of the fusion transcripts are implied as predicted by SMART [38], [39]. * denotes threshold predictions.
(XLSX)

Table S4 Primers used in the study.
(XLSX)
