# Supplementary figures and images for: Reanalysis of RNA-Sequencing Data Reveals Several Additional Fusion Genes with Multiple Isoforms
Source: PLoS One. 2012 Oct 31;7(10):e48745. doi: 10.1371/journal.pone.0048745 (PMC3485361; doi:10.1371/journal.pone.0048745)

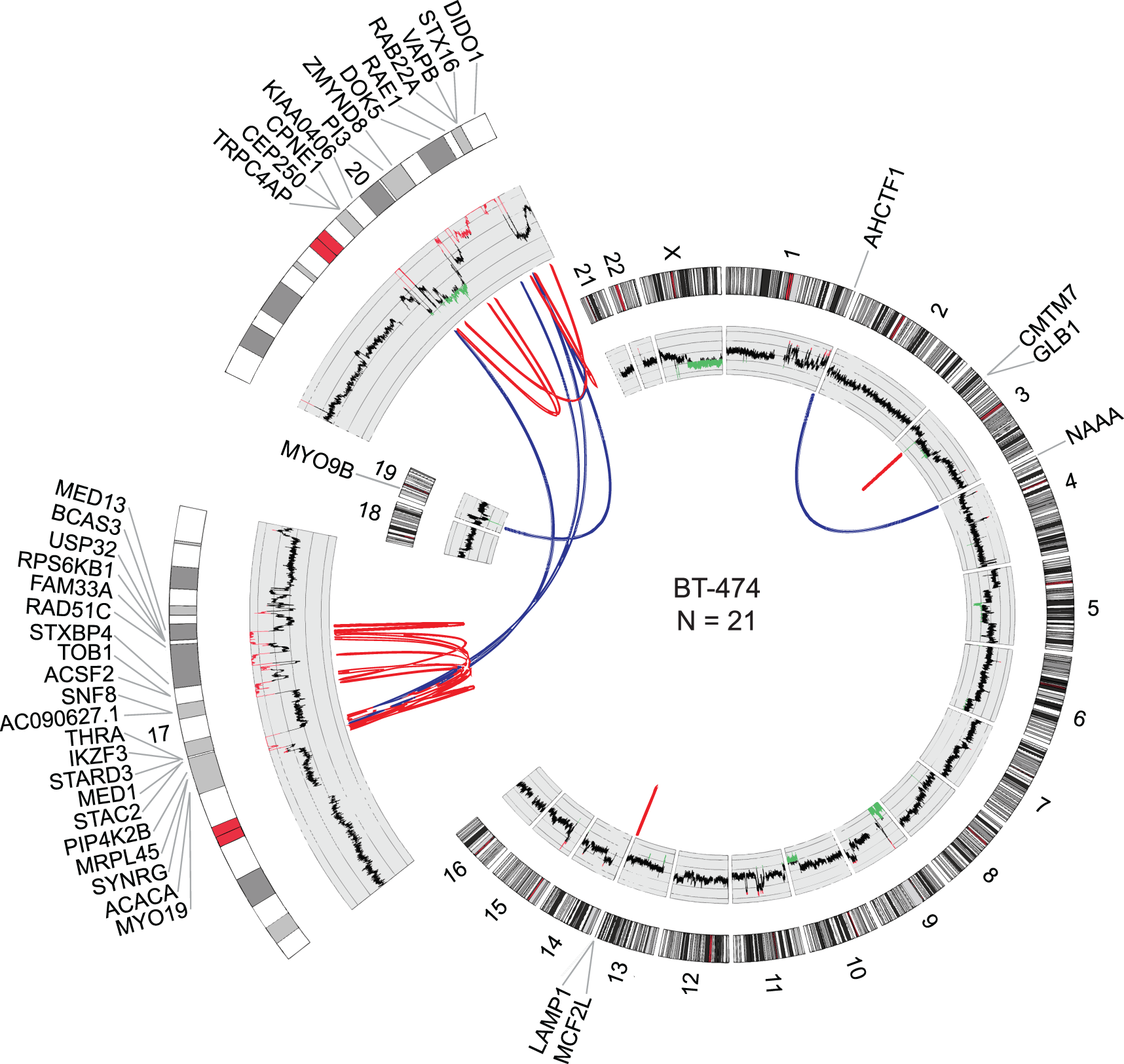

Supplement: Figure S1 — Genomic rearrangements underlying fusion gene formation in BT-474. Circos plot illustrating all chromosomal translocations in BT-474 reported by us here and previously [19]. Chromosomes are drawn into scale around the rim of the circle and data are plotted on these coordinates. Intrachromosomal (red) and interchromosomal (blue) fusions are indicated by arcs. Copy number profiles are plotted in the inner circle. Amplifications are shown in red and deletions in blue. N denotes the number of fusion genes per cell line. (TIF) [file pone.0048745.s001.tif]

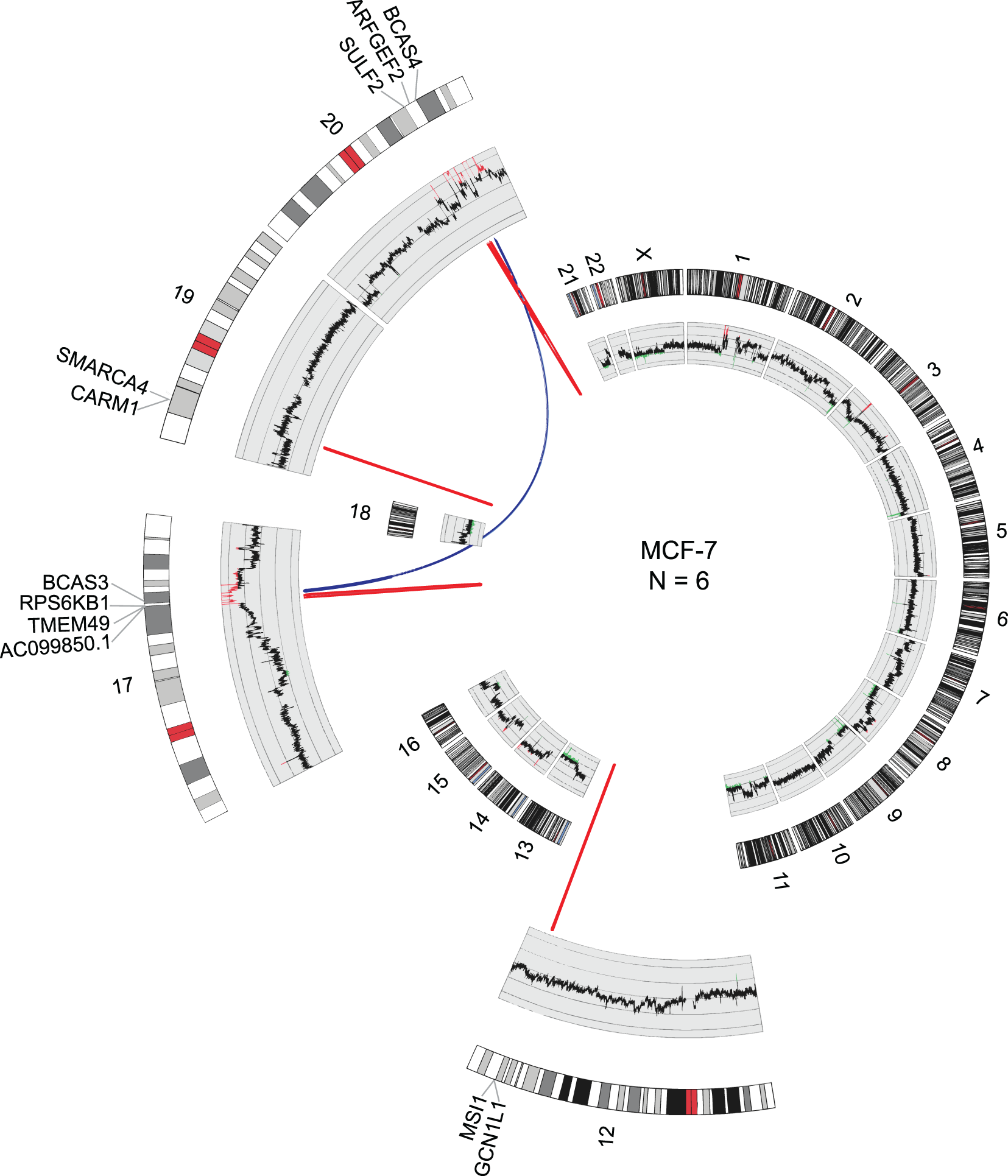

Supplement: Figure S2 — Genomic rearrangements underlying fusion gene formation in MCF-7. Circos plot illustrating all chromosomal translocations in MCF-7 reported by us here and previously [19]. Chromosomes are drawn into scale around the rim of the circle and data are plotted on these coordinates. Intrachromosomal (red) and interchromosomal (blue) fusions are indicated by arcs. Copy number profiles are plotted in the inner circle. Amplifications are shown in red and deletions in blue. N denotes the number of fusion genes per cell line. (TIF) [file pone.0048745.s002.tif]

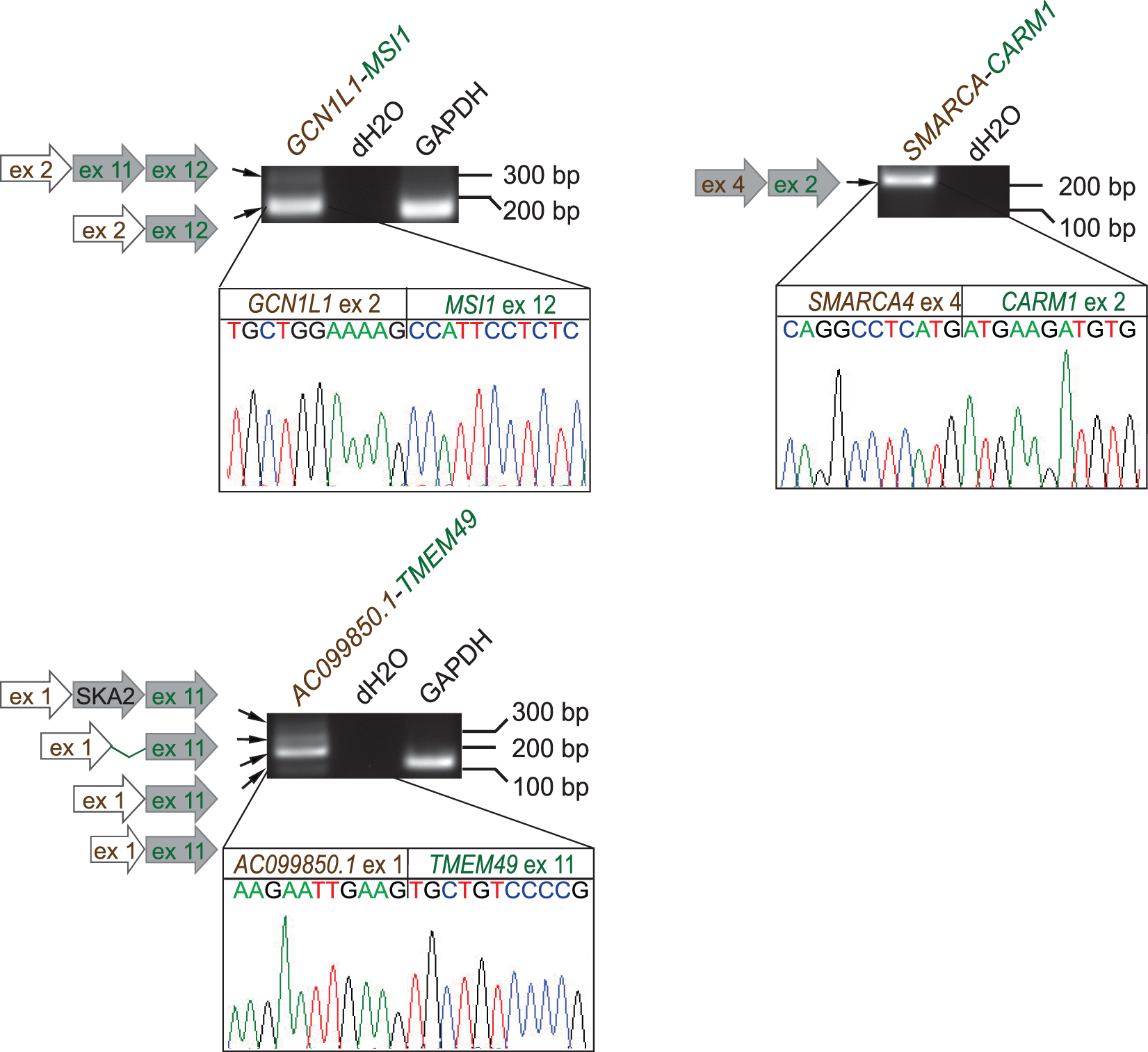

Supplement: Figure S3 — Several fusion transcripts have multiple splice variants in MCF-7. Transcript variants of MCF-7 fusion genes GCN1L1-MSI1, SMARCA4-CARM1 and AC099850.1-TMEM49 are presented. Multiple splice variants are visible as RT-PCR bands, and schematically represented by the arrows to the left. Chromatograms show the actual cDNA sequence break points of the main predicted fusion isoforms, and are connected with lines to the corresponding RT-PCR bands. Gray arrows = coding sequence, white arrows = untranslated exon or 3′/5′ UTR, thin lines connecting exons = intronic regions. 5′ partner genes are represented by brown color, 3′ partner genes by green. (TIF) [file pone.0048745.s003.tif]

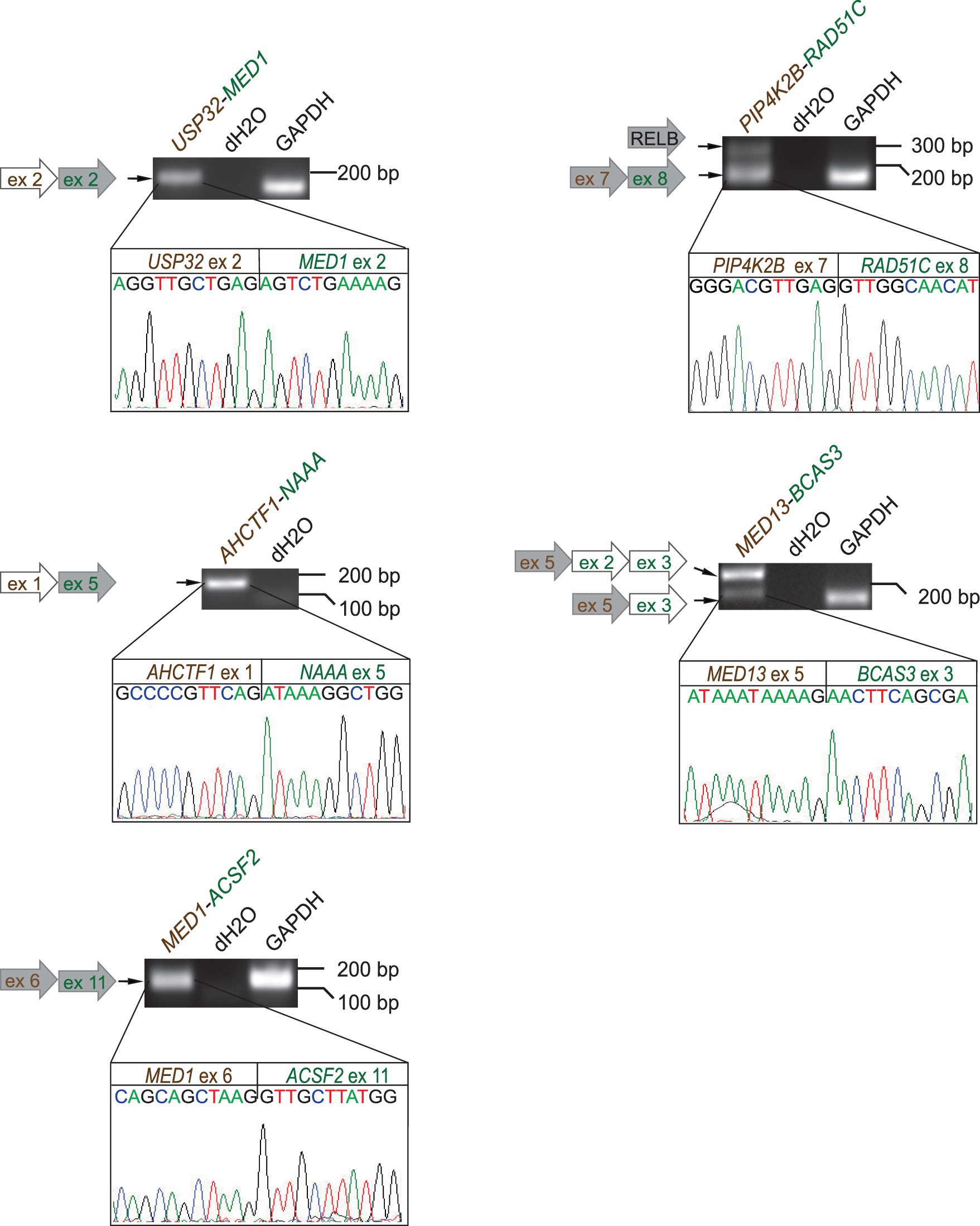

Supplement: Figure S4 — Several fusion transcripts have multiple splice variants in BT-474. Transcript variants of BT-474 fusion genes USP32-MED1, PIP4K2B-RAD51, AHCTF1-NAAA, MED13-BCAS3 and MED1-ACSF2 are presented. Multiple splice variants are visible as RT-PCR bands, and schematically represented by the arrows to the left. Chromatograms show the actual cDNA sequence break points of the main predicted fusion isoforms, and are connected with lines to the corresponding RT-PCR bands. Gray arrows = coding sequence, white arrows = untranslated exon or 3′/5′ UTR, thin lines connecting exons = intronic regions. 5′ partner genes are represented by brown color, 3′ partner genes by green. (TIF) [file pone.0048745.s004.tif]
